# Low volume polyethylene glycol combined with senna versus high volume polyethylene glycol, which regimen is better for bowel preparation for colonoscopy? A randomized, controlled, and single‐blinded trial

**DOI:** 10.1002/hsr2.829

**Published:** 2022-09-12

**Authors:** Amir Sadeghi, Khaled Rahmani, Pardis Ketabi Moghadam, Saeed Abdi, Ali Jahanian, Mobin Fathy, Mahsa Mohammadi, Mehran Mahdavi Roshan, Meysam Olfatifar, Mohammad Reza Zali, Mohammad Reza Hatamnejad, Mohsen Rajabnia

**Affiliations:** ^1^ Research Institute for Gastroenterology and Liver Diseases Shahid Beheshti University of Medical Science Tehran Iran; ^2^ Liver and Digestive Research Center Kurdistan University of Medical Sciences Sanandaj Iran; ^3^ Gastroenterology and Hepataology Diseases Research Center Qom University of Medical Sciences Qom Iran

**Keywords:** bowel preparation, colonoscopy, laxatives, polyethylene glycol, senna, side effects

## Abstract

**Background and Aims:**

Bowel preparation affects the quality of colonoscopy. Reaching the optimal preparation has been a challenge for years. Polyethylene glycol (PEG) is the sole FDA‐approved substance for this purpose. However, patients find it unpleasant and often complain about its adverse effects. In this study, we aimed to reduce these complaints by lowering the amount of PEG and adding senna which is an herbal stimulant laxative.

**Methods:**

Four hundred and eighty‐six patients were admitted for colonoscopy. Finally, 382 patients were enrolled in the study and we divided them into two groups; 186 patients were placed in which conventional high volume PEG‐alone regimen was consumed and 196 patients in which low volume PEG plus senna regimen was offered. The quality of colon preparation was compared between the two groups by independent two samples *t*‐test (or its corresponding nonparametric test), Fisher's exact, or *χ* ^2^ test in SPSS software version 22.

**Results:**

The colon preparation quality was equally efficient in the two groups as 69.36% in the high volume PEG group and 71.94% in PEG plus senna group had adequate bowel preparation (*p* = 0.58). Adverse effects, like nausea, bloating, headache, and sleeplessness were significantly less in the low volume PEG plus senna group.

**Conclusion:**

Besides the fact that bowel preparation by low volume PEG plus senna combination was noninferior to the conventional high volume PEG‐alone regimen, the side effects were much less common with the low volume PEG plus senna regimen.

## 1 INTRODUCTION

Colonoscopy is the current option for the diagnosis of many colorectal pathologies.^1^ A key element to enhance the effectiveness of colonoscopy is optimal bowel preparation.^2^ Better bowel preparation may improve colonoscopic detection of colon abnormalities, especially more diminutive ones.^3^ Moreover, better colon preparation increases the cecal intubation rate and contributes to the more diagnostic accuracy of the colonoscopy.^4^, ^5^ Thus, improvement of bowel preparation has been a rewarding challenge for physicians to date.^6^ Several factors may affect net bowel cleansing and among them, an efficacious oral preparation regimen is the main one.^7^, ^8^ Currently, the most commonly prescribed regimen consists of polyethylene glycol (PEG), a nonabsorbable polymer and osmotic laxative with a high molecular weight, formulated as a solution passing through the colon with no absorption or secretion.^9^

However, massive amounts of PEG solution required to reach the maximum efficacy, and its bad taste made it less acceptable for the patients which lead to the introduction of more easily applied regimens that contain the least amounts of PEG by adding other laxatives such as bisacodyl.^10^ Besides, these new regimens may have fewer adverse effects as one bisacodyl‐containing regimen proved to be so in a meta‐analysis, for instance.^11^ A randomized observer‐blind parallel‐group investigation conducted by Parente et al.^12^ reported no significant difference in successful cleansing between adult outpatients subjected to colonoscopy randomly allocated to 2‐L PEG‐Citrate‐Simethicone/bisacodyl or 4‐L PEG, taken as split regimens before the procedure.

There are other proposed laxatives that may ameliorate bowel preparation methods such as senna. Senna (*Cassia Angustifolia* Vahl, Leguminosae, Indian senna, Tinnevelly senna), an anthraquinone derivative, is a stimulant laxative capable of stimulating intestinal motility and affecting epithelial transport of electrolytes and water.^13^ Although senna is cost‐effective, more easily tolerated, and has fewer adverse effects, its cleansing potential remains controversial.^14^ In a study, Amato et al.^15^ investigated the efficacy of a half‐dose regimen of PEG and senna and a full‐dose regimen of senna alone. There was found to be no significant difference between the two regimens. Radaelli et al.^16^ demonstrated that overall tolerance of the preparation, the quality of colon preparation, and compliance were significantly better with the high‐dose senna regimen compared to the conventional 4‐L PEG‐electrolyte lavage solution regimen.

However, there is no consensus regarding which alternative regimen is the most appropriate replacement for the conventional 4‐L PEG regimen. It may be because of a lack of studies in this area. Here, we should determine different aspects of the high volume PEG and the combination of low volume PEG and senna to compare their efficacy in bowel cleansing.

## 2 MATERIALS AND METHODS

### 2.1 Inclusion and exclusion criteria

This study has been performed as a randomized, controlled (equivalent), single‐blinded trial between February 2021 and March 2022. It was carried out in compliance with the international instructions regarding the clinical investigation of the World Medical Association's Declaration of Helsinki; Shahid Beheshti university ethics committee approved the research (ethics code: IR. SBMU. RIGLD. REC.1400.691); Also, the investigation protocol was endorsed in the Iranian Registry of Clinical Trials with IRCT ID: IRCT20211101052935N1. All the patients signed informed consent before participation. The studied population consists of adult patients (older than 18) who require an elective colonoscopy. Four hundred and eighty‐six patients referring to the gastrointestinal clinic of Taleghani hospital for outpatient colonoscopy, were enrolled. Colonoscopy indications were screening‐related and included increased‐risk screening (cases who had one or more first‐degree relative[s] suffering from colorectal cancer [CRC] or colorectal adenomas, hereditary CRC syndromes), average‐risk screening, positive fecal occult blood test or documented iron deficiency anemia in men or postmenopausal women, lower gastrointestinal symptoms such as unexplained chronic clinically significant diarrhea, unexplained involuntary weight loss, or abnormal abdominal imaging such as abnormalities detected on barium enema, abdominal computed tomography, and so forth. A nurse clinician recruited patients for the trial during their initial assessment, before the procedure. After explaining the details of the study, the preparation regimens, possible side effects, and how to follow up on patients, all patients willing to participate in the study were informed.

**Figure 1:**
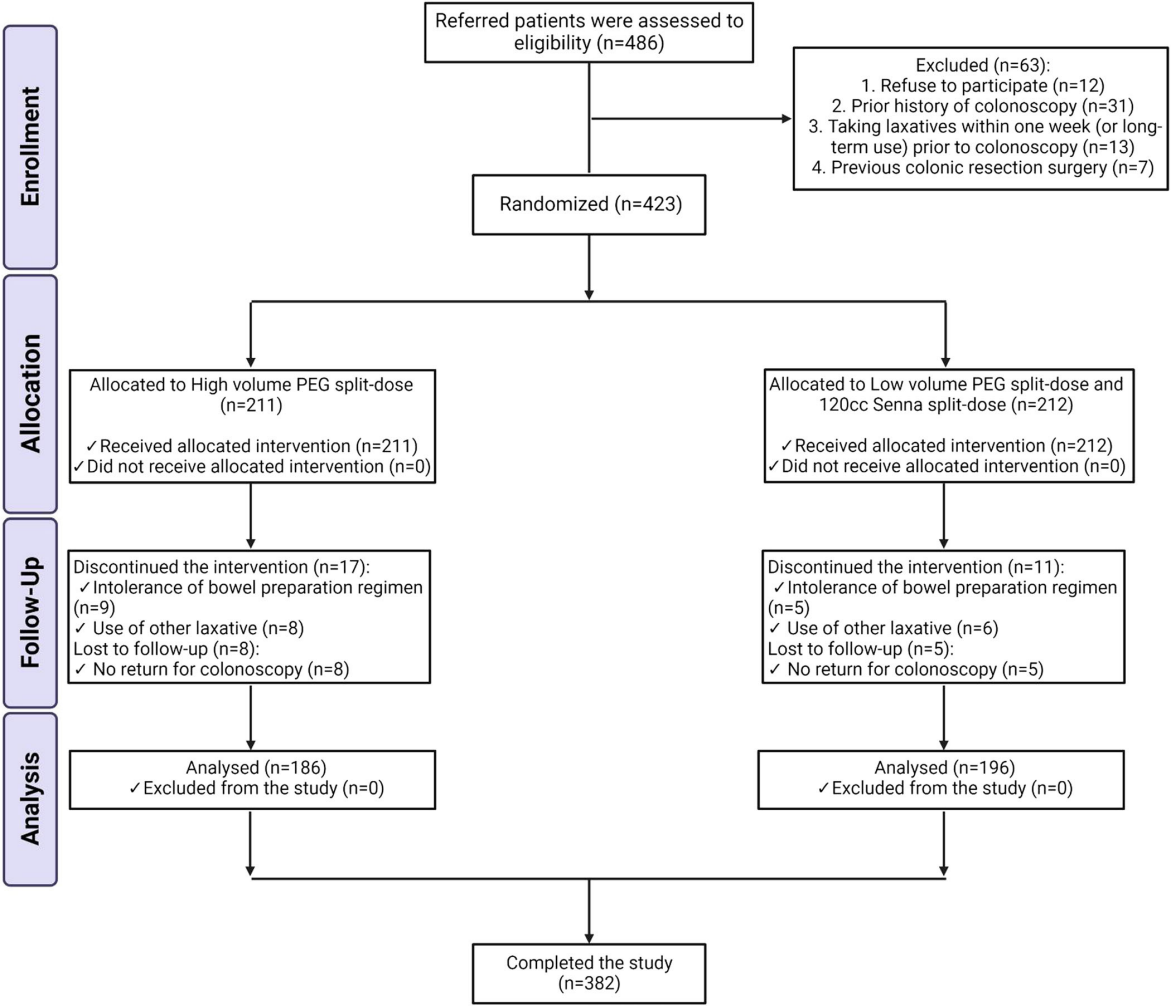
Consort flow diagram of the study. PEG, polyethylene glycol.

The exclusion criteria included^1^ allergy to PEG or senna^2^; intolerance to bowel preparation regimen^3^; failure to fully comply with the bowel preparation regimen (less than 5 PEG powder sachet and/or 120 ml senna solution)^4^; patients taking laxatives within 1 week before enrollment^5^; patients who had previous colonic resection surgery^6^; known or suspected bowel obstruction^7^; prior histories of colonoscopy. Before entering the study, 63 patients were excluded. Twelve patients left the study due to refusal to participate. Thirty‐one patients were excluded due to prior history of colonoscopy, 13 patients due to taking laxatives within 1 week (or long‐term use) before colonoscopy, and 7 patients due to previous colon resection surgeries (Figure 1).

### 2.2 Randomization

Among 423 patients left in our study, randomization was performed. The method of random allocation was the formation of permuted block randomization. If you consider the bowel preparation regimen of high volume PEG as A and the combination of low volume PEG and senna as B, patients were prepared for colonoscopy according to the following pattern (randomly selected from all possible sextet blocks): Block 1: AAABBB, Block 2: BBBAAA, and so forth.

A nurse (study coordinator) allocated patients to their groups and guided them regarding the appropriate use of their considered bowel preparation method. Two hundred and eleven cases were placed in the PEG‐alone group but 25 patients were omitted later due to intolerance to bowel preparation regimen (9 patients), not returning for colonoscopy (8 patients), and the use of other laxatives (8 patients). One hundred and eighty‐six patients were left in the PEG‐alone group. On the other hand, 212 patients were placed in the PEG‐senna group which had senna in their regimen, but 16 patients were excluded later due to intolerance to bowel preparation regimen (5 patients), not returning for colonoscopy (5 patients), and the use of other laxatives (6 patients). Eventually, 196 patients were left in the PEG‐senna group (Figure 1).

### 2.3 Administration of regimens

In this study, all patients underwent a colonoscopy in the morning and we used a split‐dose preparation, meaning administration of half of the colon cleansing agents the evening before the colonoscopy and the second half the morning of the colonoscopy (5 h before the procedure; of course, by observing fasting at least 2 h before the procedure).

The subjects were advised to have a low‐residue diet for 4 days and only clear fluids for at least 1 day before the colonoscopy and receive food list pamphlets to eat and avoid.

The subjects of the control group (high volume PEG) received 4 L of PEG in divided doses. Therefore, 4 L of solution were consumed in divided doses from 4 p.m. of the day before the colonoscopy to 5 h before the procedure. The patients took each liter of the solution within 1 h (250 ml every 10–15 min). Approximately one‐and‐a‐half‐hour interval between each 1 L was required.

The patients of the case group (low volume PEG), received 2 L PEG, as the way mentioned above, combined with the senna solution (120 ml) in two divided doses: 60 ml at 4 p.m. the day before the procedure and 60 ml at the morning of the colonoscopy (5 h before the procedure).

To avoid bias, all colonoscopies were performed by two endoscopists unaware of the bowel preparation regimens of the patients. Both endoscopists were present during colonoscopies evaluating bowel preparation simultaneously. Patients' questions about the preparation were answered by the study coordinator to prevent unblinding the endoscopists. Colonoscopies were recorded and reviewed by other endoscopists for re‐evaluation of bowel preparation. Videos of colonoscopies were reviewed by performing endoscopists and two other specialists for a finalized bowel preparation scoring that all of them agreed upon.

### 2.4 Data collection

Shortly before colonoscopy and after obtaining informed consent from the patient, the patients were interviewed for the assessment of demographic features such as gender (female, male), age (year), education level, medical history, drug history, acceptance, and side effects of the preparation agents. Additionally, the patient's weight (kg) and height (m) were evaluated to determine body mass index (BMI) (kg/m^2^).

The BBPS was applied as an objective and validated means of evaluating bowel preparation. BBPS involves assigning a score from 0 to 3 to each of the right side, transverse section, and left side of the colon. The scores are summed for a total BBPS score, ranging from 0 (poor) to 9 (excellent)^1^: Score 0: Unprepared colon with mucosa not seen due to solid stool with no possibility of clearance^2^; Score 1: Portion of the colon mucosa can be observed, but other regions of the colon segment not seen appropriately due to residual stool, staining, and/or opaque liquid; ^3^ Score 2: Minor amount of residual staining, small portions of opaque liquid and/or stool, but the vision of most of the mucosa is satisfactory^4^; Score 3: Whole colon mucosa can be observed well without residual staining, small fragments of stool, and/or opaque liquid. Scores were noted in a form by endoscopists, at the time of withdrawal, after the measures to improve visualization (i.e., wash or suction). In addition, endoscopists recorded cecal intubation time, withdrawal time, and the possible polyp number and site.

### 2.5 Statistical analysis

First data were summarized using percentage, frequency, mean and standard deviation. To compare the quantitative variables between the two groups under, independent two samples *t*‐test or Mann–Whitney *U*‐test as its nonparametrical equivalent was used, and to measure the distribution of qualitative variables between the two groups, Fisher exact or *χ* ^2^ tests were applied. The results were presented at a statistical significance of 0.05 and using two‐tailed tests. All analyses were performed in SPSS 22.

## 3 RESULTS

Of 382 included patients, 186 patients received the PEG‐alone regimen and 196 patients received the PEG‐senna regimen. The baseline demographic features of the patients are presented in Table 1.

**Table 1:** Characteristics of the study population

	High volume PEG (*n* = 186)	Low volume PEG‐senna (*n* = 196)	*p* Value
Age (year)	49.47 ± 11.12	48.88 ± 11.75	0.13^a^
Gender			
Female	102 (54.84%)	108 (55.10%)	0.96^b^
Male	84 (45.16%)	88 (44.90%)	
Education			
Illiterate	13 (7.0%)	17 (8.67%)	0.091^b^
Nonacademic education	116 (62.36%)	138 (70.41%)	
Academic education	57 (30.64%)	41 (20.92%)	
BMI (kg/m^2^)	26.34 ± 4.18	26.48 ± 3.44	0.84^a^
Waist/hip	0.96 ± 0.06	0.96 ± 0.05	0.96^c^
Smoking	27 (14.52%)	26 (13.27%)	0.72^b^
Opium	15 (8.06%)	17 (8.67%)	0.83^b^

Abbreviations: BMI, body mass index; PEG, polyethylene glycol.

^a^ Independent two samples *t*‐test.

^b^ *χ* ^2^ test.

^c^ Mann–Whitney *U*‐test.

The mean age in the PEG‐alone and PEG‐senna groups was 49.47 ± 11.12 and 48.88 ± 11.75, respectively. Female patients were more populous than male patients in both groups. Most of the patients had nonacademic education as more than half of them in both groups are placed in this category. The mean ± SD BMI and waist/hip ratio in both groups are near to the total and almost equal to each other. Also, we asked about patients' drug history including constipating drugs such as opiates, iron supplements, and so forth, and there was no meaningful difference between the two groups in this category.

The conditions that lead the patients to colonoscopy are shown in Table 2. In the case of the total population of the patients, average‐risk CRC screening was the most frequent indication for colonoscopy followed by iron deficiency anemia, a positive fecal occult blood test, unexplained abdominal pain, involuntary weight loss, chronic constipation, a family history of CRC, and chronic diarrhea in respective order. These results were almost the same in both PEG‐alone and PEG‐senna groups.

**Table 2:** Indications of colonoscopy

Indications of colonoscopy	Total (*n* = 382)	High volume PEG (*n* = 186)	Low volume PEG‐senna (*n* = 196)	*p* Value^a^
Average‐risk CRC screening	94 (24.60%)	46 (24.73%)	48 (24.49%)	0.96
Positive fecal occult blood	90 (23.56%)	43 (23.12%)	47 (24.00%)	0.84
Iron deficiency anemia	62 (16.23%)	30 (16.12%)	32 (16.32%)	0.96
Unexplained abdominal pain	43 (11.26%)	22 (11.83%)	21 (10.71%)	0.73
Involuntary weight loss	28 (7.33%)	13 (7.00%)	15 (7.65%)	0.80
Chronic constipation	26 (6.81%)	14 (7.52%)	12 (6.12%)	0.59
Family history of CRC	22 (5.76%)	10 (5.38%)	12 (6.12%)	0.75
Chronic diarrhea	17 (4.45%)	8 (4.30%)	9 (4.59%)	0.89

Abbreviations: CRC, colorectal cancer; PEG, polyethylene glycol.

^a^ *χ* ^2^ test.

One of the major aims of our research was to compare the bowel cleansing potential of a PEG‐senna regimen combined with senna to that of the conventional, FDA‐approved PEG‐alone regimen. The intestinal preparation of each segment and the whole colon is demonstrated in Table 3.

**Table 3:** Comparison of bowel preparation between two groups (BBPS)

Colonic segments	BBPS	Total	High volume PEG (*n* = 186)	Low volume PEG‐senna (*n* = 196)	*p* Value^a^
Left colon	Excellent	38 (9.95%)	18 (9.68%)	20 (10.20%)	0.59
	Good	270 (70.68%)	128 (68.82%)	142 (72.45%)	
	Poor/inadequate	74 (19.37%)	40 (21.50%)	34 (17.35%)	
Transverse colon	Excellent	47 (12.31%)	21 (11.29%)	26 (13.27%)	0.72
	Good	282 (73.82%)	137 (73.66%)	145 (73.98%)	
	Poor/inadequate	53 (13.87%)	28 (15.05%)	25 (12.75%)	
Right colon	Excellent	44 (11.52%)	19 (10.21%)	25 (12.76%)	0.73
	Good	244 (63.87%)	120 (64.52%)	124 (63.26%)	
	Poor/inadequate	94 (24.61%)	47 (25.27%)	47 (23.98%)	
Total colon	Excellent	31 (8.11%)	13 (6.99%)	18 (9.18%)	0.67
	Good	239 (62.57%)	116 (62.37%)	123 (62.76%)	
	Poor/inadequate	112 (29.32%)	57 (30.64%)	55 (28.06%)	

Abbreviations: BBPS, Boston bowel preparation scale; PEG, polyethylene glycol.

^a^ *χ* ^2^ test.

Left colon preparation was found to be inadequate (BBPS = 0 or 1) in 74 patients (19.37%), good (BBPS = 2) in 270 patients (70.68%), and excellent (BBPS = 3) in 38 patients (9.95%). Among patients who received the PEG‐alone regimen, left colon preparation was inadequate in 40 patients (21.50%), good in 128 patients (68.82%), and excellent in 18 patients (9.68%). On the other side, among patients who received the PEG‐senna regimen combined with senna, left colon preparation was inadequate in 34 patients (17.35%), good in 142 patients (72.45%), and excellent in 20 patients (10.20%). Therefore, the PEG‐senna regimen appeared to be almost equal to the PEG‐alone regimen in intestinal cleansing of the left side of the colon.

Transverse colon preparation was inadequate in 53 patients (13.87%), good in 282 patients (73.82%), and excellent in 47 patients (12.31%). Among patients who received the PEG‐alone regimen, transverse colon preparation was inadequate in 28 patients (15.05%), good in 137 patients (73.66%), and excellent in 21 patients (11.29%). On the other side, among patients who received the combined PEG‐senna regimen, transverse colon preparation was inadequate in 25 patients (12.75%), good in 145 patients (73.98%), and excellent in 26 patients (13.27%). Thus, the PEG‐senna regimen seems to be equally sufficient compared to the PEG‐alone regimen in bowel cleansing of the transverse colon as it has almost the same inadequate, good, and excellent preparation rate.

Right colon preparation was inadequate in 94 patients (24.61%), good in 244 patients (63.87%) and excellent in 44 patients (11.52%). Among patients who received the PEG‐alone regimen, right colon preparation was inadequate in 47 patients (25.27%), good in 120 patients (64.52%), and excellent in 19 patients (10.21%). On the other side, among patients who received the PEG‐senna regimen containing senna, right colon preparation was inadequate in 47 patients (23.98%), good in 124 patients (63.26%), and excellent in 25 patients (12.76%). Therefore, like the two other segments mentioned above, the PEG‐senna regimen seems to be equally potent compared to the PEG‐alone regimen in the intestinal preparation of the right side of the colon.

Total colon preparation was found to be inadequate (BBPS = 0‐5) in 112 patients (29.32%), good (BBPS = 6 or 7) in 239 patients (62.57%), and excellent (BBPS = 8 or 9) in 31 patients (8.11%). Among patients who received the PEG‐alone regimen, total colon preparation was inadequate in 57 patients (30.64%), good in 116 patients (62.37%), and excellent in 13 patients (6.99%). On the other side, among patients who received the PEG‐senna regimen combined with senna, total colon preparation was inadequate in 55 patients (28.06%), good in 123 patients (62.76%), and excellent in 18 patients (9.18%). Hence, there might be a mild improvement in total bowel preparation with the PEG‐senna regimen but it was not a significant difference.

**Figure 2:**
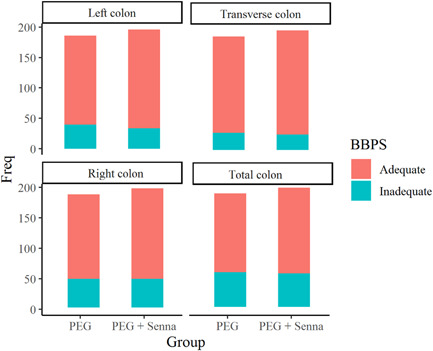
Comparison of bowel preparation between two groups (BBPS). There is no significant, meaningful difference between the two regimens in their ability of bowel cleansing. BBPS, Boston bowel preparation scale; PEG, polyethylene glycol.

As mentioned before, adequate intestinal preparation enhances reliable findings during colonoscopy. Adequate preparation is defined as a BBPS of six or more. Data regarding the adequacy of bowel preparation in our study is shown in Table 4 and Figure 2.

**Table 4:** Comparison of bowel preparation between two groups (BBPS)

Colonic segments	BBPS	Total	High volume PEG (*n* = 186)	Low volume PEG‐senna (*n* = 196)	*p* Value^a^
Left colon	Adequate	308 80.63%)	146 (78.50%)	162 (82.65%)	0.30
	Inadequate	74 (19.37%)	40 (21.50%)	34 (17.35%)	
Transverse colon	Adequate	329 (86.13%)	158 (84.95%)	171 (87.25%)	0.52
	Inadequate	53 (13.87%)	28 (15.05%)	25 (12.75%)	
Right colon	Adequate	288 (75.39%)	139 (74.73%)	149 (76.02%)	0.77
	Inadequate	94 (24.61%)	47 (25.27%)	47 (23.98%)	
Total colon	Adequate	270 (70.68%)	129 (69.36%)	141 (71.94%)	0.58
	Inadequate	112 (29.32%)	57 (30.64%)	55 (28.06%)	

Abbreviations: BBPS, Boston bowel preparation scale; PEG, polyethylene glycol.

^a^ *χ* ^2^ test.

Among 186 cases who received the PEG‐alone regimen, 146 patients (78.50%) had adequate preparation in the left colon; whereas among 196 patients who received the PEG‐senna regimen, 162 patients (82.65%) reached adequate left colon preparation. Moreover, in the transverse colon of 158 cases in the PEG‐alone group (84.95%) and 171 cases in the PEG‐senna group (87.25%), adequate preparation could be seen. However, differences between the two groups in the right colon and total colon preparation were less significant than in the other two segments; but still, the PEG‐senna regimen appeared slightly superior and more potent in bowel cleansing. As shown in Table 4, 139 patients of the PEG‐alone group (74.73%) had adequate right colon preparation, compared to 149 patients of the PEG‐senna group (76.02%). Also, 129 patients of the PEG‐alone group (69.36%) had adequate total colon preparation, whereas 141 patients of the PEG‐senna group (71.94%) reached adequate total colon preparation which shows that the PEG‐senna regimen slightly improved the rate of successful colonoscopy, but these changes are not meaningful.

The colonoscopy features of our study are exhibited in Table 5. In total, nine patients (2.36%) had incomplete colonoscopy due to insufficient intestinal preparation, of which five of them were in the PEG‐alone group (2.69% out of 186 patients) and four of them were in the PEG‐senna group (2.04% out of 196 patients). Other patients had successful cecal intubation. In the total population, mean ± SD cecal intubation time was 4:12 ± 1:22 (ranging from 1:40 to 8:38) whereas in the PEG‐alone group it was 4:16 ± 1:15 (ranging from 2:04 to 8:38) and in the PEG‐senna group it was 4:08 ± 1:24 (ranging from 1:40 to 8:09). Additionally, total mean ± SD withdrawal time was 4:29 ± 1:25 (ranging from 1:18 to 9:02) whereas in the PEG‐alone group it was 4:45 ± 1:11 (ranging from 1:18 to 7:36) and in the PEG‐senna group it was 4:23 ± 1:31 (ranging from 1:32 to 9:02). Collectively, mean ± SD total colonoscopy time was 8:48 ± 2:06 in total population (ranging from 3:42 to 16:28) whereas in the PEG‐alone group it was 8:58 ± 1:52 (ranging from 3:56 to 12:58) and in the PEG‐senna group it was 8:36 ± 2:12 (ranging from 3:42 to 16:28). These data show that there is no significant difference between the two groups regarding their ability to shorten the procedure duration.

**Table 5:** Colonoscopy parameters

Colonoscopy parameters	Total	High volume PEG	Low volume PEG‐senna	*p* Value
Premature withdrawal due to insufficient bowel preparation (Incomplete colonoscopy), *n* (%)	9/382 (2.36%)	5/186 (2.69%)	4/196 (2.04%)	0.83^a^
Cecal intubation rate, *n* (%)	373/382 (97.64%)	181/186 (97.31%)	192/196 (97.96%)	0.68^a^
Cecal insertion time (min), mean ± SD (95% CI for mean)	4.12 ± 1:22 (1.40–8.38)	4.16 ± 1.15 (2.04–8.38)	4.08 ± 1.24 (1.40–8.09)	0.91^b^
Withdrawal time (min), mean ± SD (95% CI for mean)	4.29 ± 1.25 (1.18–9.02)	4.45 ± 1.11 (1.18–7.36)	4.23 ± 1.31 (1.32–9.02)	0.89^b^
Total colonoscopy time (min), mean ± SD (95% CI for mean)	8.48 ± 2.06 (3.42–16.28)	8.58 ± 1.52 (3.56–12.58)	8.36 ± 2.12 (3.42–16.28)	0.87^b^
Polyp detection rate, *n* (%)	89 (23.30%)	34 (18.28%)	55 (28.06%)	**0.024** c
Adenoma detection rate, *n* (%)	45 (11.78%)	18 (9.68%)	27 (13.78%)	0.21^c^

*Note*: Bold value is significant <0.05.

Abbreviations: CI, confidence interval; BBPS, Boston bowel preparation scale; PEG, polyethylene glycol; SD, standard deviation.

^a^ Fisher's exact test.

b Independent two samples *t*‐test,

c *χ* ^2^ test.

Out of 382 patients, 89 patients (23.30%) were detected to have polyps and 45 patients (11.78%) had adenomas in their colon. Among 186 patients who received the PEG‐alone regimen, 34 patients (18.28%) were detected with polyps and 18 patients (9.68%) had adenomas; whereas among 196 patients who received the PEG‐senna regimen, 55 patients (28.06%) were detected with polyps and 27 patients (13.78%) had adenomas. These results imply that low‐PEG senna‐containing regimen may improve our ability to detect polyps and, to a lesser extent, adenomas.

**Figure 3:**
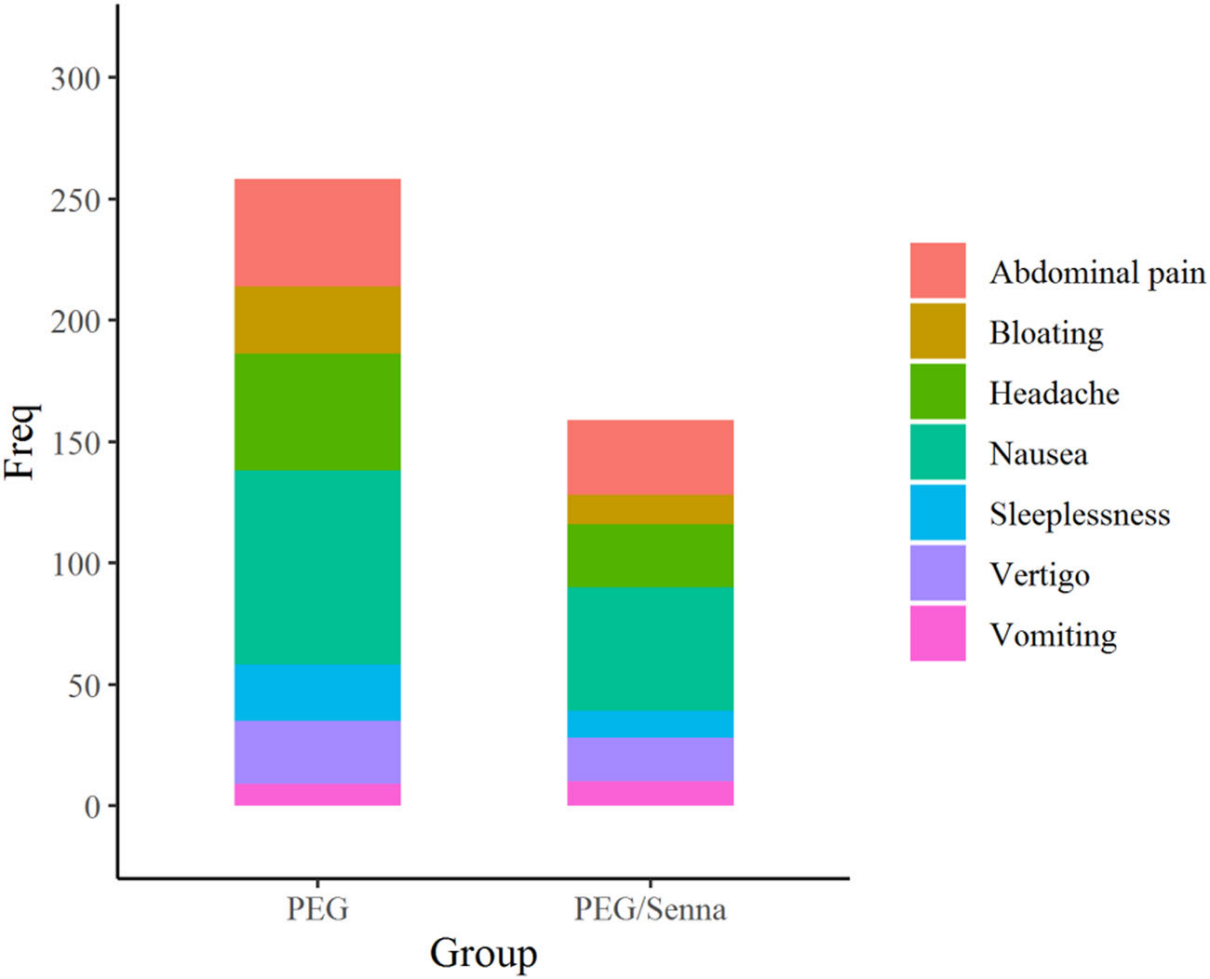
Frequency of adverse effects of the two regimens. Overally, low volume PEG plus senna regimen has fewer adverse effects compared to high volume PEG alone regimen. Most of the adverse effects are clearly less frequent in the PEG plus senna group. PEG, polyethylene glycol.

Records of side effects caused by preparation regimens are brought in Table 6 and Figure 3. Nausea was the most frequent adverse effect of both regimens affecting 80 cases in the PEG‐alone group (42.63%) and 51 cases in the PEG‐senna group (27.80%). Abdominal pain was the second most common adverse effect in the total population affecting 75 patients (18.90%). Besides, 44 patients of the PEG‐alone group (21.82%) and 31 patients of the PEG‐senna group (14.63%) had abdominal pain following the consumption of the regimens.

**Table 6:** Evaluation of the ease of preparation by patients

Side effects	Total (382)	High volume PEG (*n* = 186)	Low volume PEG‐senna (*n* = 196)	OR (CI)	*p* Value^a^
Nausea, *n* (%)	131 (35.07%)	80 (42.63%)	51 (27.80%)	1.99 (1.27–3.16)	**<0.001**
Abdominal pain, *n* (%)	75 (18.90%)	44 (21.82%)	31 (14.63%)	1.65 (0.96–2.85)	0.054
Headache, *n* (%)	74 (18.15%)	48 (23.85%)	26 (14.14%)	2.27 (1.30–4.02)	**0.002**
Vertigo, *n* (%)	44 (12.43%)	26 (14.21%)	18 (10.73%)	1.61 (0.81–3.23)	0.142
Bloating, *n* (%)	40 (11.19%)	28 (16.75%)	12 (5.85%)	2.72 (1.28–6.06)	**0.004**
Sleeplessness, *n* (%)	34 (7.96%)	23 (11.16%)	11 (4.87%)	2.37 (1.07–5.55)	**0.021**
Vomiting, *n* (%)	19 (4.97%)	9 (4.56%)	10 (5.36%)	0.95 (0.33–2.66)	0.910

*Note*: Bold values are significant <0.05.

Abbreviations: CI, confidence interval; OR, odds ratio; PEG, polyethylene glycol.

^a^ *χ* ^2^ test.

Headache was almost as common as abdominal pain affecting 74 patients (18.15%) in the total population. Moreover, 48 patients of the PEG‐alone group (23.85%) and 26 patients of the PEG‐senna group (14.14%) experienced abdominal pain during preparation. Other side effects including vertigo, bloating, insomnia, and vomiting are listed in Table 6.

All these side effects except vomiting occurred less frequently with the consumption of the PEG‐senna regimen. Thus, the addition of senna to the preparation regimen may reduce the need for PEG and ultimately reduce the adverse effects of preparation without jeopardizing the quality of bowel preparation.

## 4 DISCUSSION

The importance of screening for CRC using colonoscopy as a superior strategy has been raised in the literature.^17^ But, when it comes to colonoscopy, there lies a big challenge regarding colon preparation. A colonoscopy that suffers from poor preparation will increase the risk of missed polyps, prolongation of cecal intubation time, and the need for recolonoscopy as well as adverse events.^18^ Although an adequate preparation is strongly related to the patient's characteristics such as quality of routine bowel movements, having certain comorbidities interfering with normal bowel movements such as diabetes and dementia, taking medications or illicit drugs inducing constipation like opioids, ferrous sulfate, and tricyclic antidepressants,^19^ preparation solutions and prescription schedules are also important factors.^20^ PEG which is mixed with an iso‐osmolar electrolyte solution has been accepted worldwide due to its safety in fluid and electrolyte balance as well as its ability to irrigate the colon.^21^ Some of the reported side effects such as exacerbation of heart failure, aspiration, Mallory‐Weiss tear, hyponatremia, and patient's dissatisfaction are attributed to the need for consumption of at least 4 L of the mentioned solution to reach an acceptable preparation.^9^ To overcome the obstacle of overhydration, adding stimulants or osmotic laxatives has been suggested. However, the adverse events of stimulant agents like bisacodyl and osmotic agents like sodium phosphate which are respectively ischemic colitis and dangerous electrolyte imbalance should be considered.^22^, ^23^ Another introduced alternative to this problem is splitting the liquid in half between the evening of the day before and the morning of the day of colonoscopy in contrast to nonseparated consumption of the preparation solution the day before the colonoscopy.^24^ Some clinicians suggest patients add some flavors to the solution to make it tasty.^25^ The moot point is a 5‐h interval between the last dose of liquid ingestion and the time of colonoscopy which is advised to be respected for both methods of administration.^26^ Although there are some studies comparing the effectiveness and side effects of various lavage solutions with standard PEG, there is still some room for further studies in this field to provide patients with more potent and tolerable low volume combinations for colon preparation. In this study, we have attempted to compare the quality of colon preparation and adverse events of two methods of colonic lavage in patients referred to the clinic and endoscopic ward of Taleghani hospital, Tehran, Iran, for colonoscopy. The focus of our investigation is on introducing a low volume solution with acceptable cleansing power and tolerable side effects. The importance of such preparation solutions is notably manifested for Inflammatory bowel disease patients who are not allowed to use high volume PEG during flares.^27^

Our results provide strong evidence that excellent and good preparations in transverse, right, and total colon are comparable between the two groups of this study. There is only a statistical difference between the preparation quality of the left colon in patients receiving the PEG‐alone regimen and the PEG‐senna regimen. As indicated in our results, excellent and good preparations in the left colon are significantly more in the PEG‐senna group (*p* < 0.05). However, we could not claim that this difference is compelling to introduce PEG‐senna lavage solutions more efficacious than PEG‐alone solutions since total colon preparation has not been affected by the superior results of left colon preparation. The comparison of the interesting outcomes between the two study groups did not confirm the findings of other previous studies, namely the study of Radaelli et al.^16^ and Vradelis et al.^28^ which strongly introduce senna as a more efficient alternative to standard PEG‐ELS. Bowel preparation in each third of the colon is not generally supposed to be different among various investigations like the pilot study of Yenidogan et al.^29^ which introduces senna alkaloids as a safe and effective additive to preparation solutions for all three parts of the colon.^30^ As indicated in our study, premature withdrawal due to cecal intubation rate, inadequate preparation, cecal intubation time, and total colonoscopy time were not affected by using different methods of preparation. Given our findings, there was no significant relationship between the type of preparation and positive colonoscopic findings such as adenoma detection rate that is comparable to the results of the study of Vradalis et al.^28^ On the contrary, the polyp detection rate is higher in low‐PEG senna‐containing group. However, this statistically significant result does not imply clinical significance, and further attempts are required to remove any confounding factors such as the heterogenicity of the two groups.

The results showed that using the low volume PEG‐senna protocol has significantly decreased the probability of the standard PEG side effects which are commonly nausea, headache, abdominal pain, bloating, and sleeplessness. These results except for abdominal pain are in line with other studies comparing the side effects of senna with conventional PEG solutions. Surprisingly, our findings indicated that abdominal pain is also found to be less common in the PEG‐senna group which was shown to be more severe and more prevalent in the PEG‐senna group in some previous studies (Shavakhi and colleagues). Additionally, our findings demonstrated improvement in some symptoms such as nausea, vomiting, and abdominal cramps due to the addition of senna to the colon preparation. It should be noted that improvement in nausea and vomiting resulting from senna addition is consistent with studies of Shavakhi and colleagues. while results of this study about improvement in abdominal cramps are not consistent with those two studies.^31^, ^32^ According to our data, the PEG‐senna regimen can emerge as a reasonable approach for colon preparation before elective colonoscopy since it can reduce the rate of overall adverse events and improve patient overall acceptance along with the fact that its efficiency in quality of preparation is at least noninferior to the standard regimen. Our study was performed in only one clinical establishment and larger studies in more than one center and region are required to confirm our findings. Another issue with our study is that the patients with past medical conditions were excluded and studies are necessary to examine our results in these patients. Additionally, it is suggested that, unlike our single‐blinded study, future studies in this field be performed as double‐blinded investigations to eliminate any kind of bias that may occur.

## 5 CONCLUSION

Colonoscopy is the main strategy for CRC screening and is also an important method of diagnosis and treatment of endoluminal colon lesions. Inadequate preparedness for colonoscopy can lead to the missed diagnosis of lesions with great potential for future growth and becoming malignant. A proper regimen for colonoscopy preparation is the key to providing the best level of colon visualization with the least discomfort. The findings of this study indicate a noninferiority trial introducing a low volume PEG‐senna regimen for colon preparation compared with a standard high volume PEG regimen pointing to the fewer side effects of the introduced regimen.

## AUTHOR CONTRIBUTIONS

**Amir Sadeghi**: Conceptualization; data curation; funding acquisition; investigation; project administration; supervision; writing – review & editing. **Khaled Rahmani**: Formal analysis; methodology; software; validation. **Pardis Ketabi Moghadam**: Data curation; validation; visualization; writing – review & editing. **Saeed Abdi**: Data curation; funding acquisition; investigation; project administration; resources. **Ali Jahanian**: Data curation; investigation; project administration; visualization; writing – original draft; writing – review & editing. **Mobin Fathy**: Data curation; investigation; validation; visualization. **Mahsa Mohammadi**: Data curation; investigation; writing – review & editing. **Mehran Mahdavi Roshan**: Data curation; investigation; visualization; writing – original draft. **Meysam Olfatifar**: Data curation; investigation; visualization; writing – review & editing. **Mohammad Reza Zali**: Conceptualization; funding acquisition; investigation; project administration; supervision; validation; visualization; writing – review & editing. **Mohammad Reza Hatamnejad**: Formal analysis; software; visualization; writing – review & editing. **Mohsen Rajabnia**: Conceptualization; data curation; formal analysis; funding acquisition; investigation; methodology; project administration; resources; software; supervision; validation; visualization; writing – original draft; writing – review & editing. All authors have read and approved the manuscript's final version.

## CONFLICT OF INTEREST

The authors declare no conflict of interest.

## TRANSPARENCY STATEMENT

The lead author Mohsen Rajabnia affirms that this manuscript is an honest, accurate, and transparent account of the study being reported; that no important aspects of the study have been omitted; and that any discrepancies from the study as planned (and, if relevant, registered) have been explained.

## Data Availability

All data generated or analyzed during this study are included in this article and its Supporting Information files data and can be requested from the corresponding author. Mohsen Rajabnia had full access to all data and takes complete responsibility for the data integrity and the accuracy of the data analysis.
